# A pragmatic, multicentre, randomised controlled trial comparing stapled haemorrhoidopexy to traditional excisional surgery for haemorrhoidal disease (eTHoS): study protocol for a randomised controlled trial

**DOI:** 10.1186/1745-6215-15-439

**Published:** 2014-11-11

**Authors:** Angus J M Watson, Hanne Bruhn, Kathleen MacLeod, Alison McDonald, Gladys McPherson, Mary Kilonzo, John Norrie, Malcolm A Loudon, Kirsty McCormack, Brian Buckley, Steven Brown, Finlay Curran, David Jayne, Ramesh Rajagopal, Jonathan A Cook, On behalf of the eTHoS study group

**Affiliations:** Department of Surgery, Raigmore Hospital, Old Perth Road, Inverness, IV2 3UJ UK; Centre for Healthcare Randomised Trials, University of Aberdeen, Health Sciences Building, Foresterhill, Aberdeen, AB25 2ZD UK; Health Economics Research Unit, University of Aberdeen, Polwarth Building, Foresterhill, Aberdeen, AB25 2ZD UK; NHS Grampian, Department of Surgery, Aberdeen Royal Infirmary, Foresterhill Road, AB25 2ZN Aberdeen, UK; Department of Surgery, Philippine General Hospital, University of the Philippines, Manila, Philippines; Sheffield Teaching Hospitals NHS Foundation Trust, Department of Surgery, Northern General Hospital, Herries Road, Sheffield, S5 7AU UK; Department of Surgery, Manchester Royal Infirmary, Central Manchester University Hospitals NHS Foundation Trust, Oxford Road, Manchester, M13 9WL UK; The Leeds Teaching Hospitals NHS Trust, St James’ Hospital, Beckett Street, Leeds, West Yorkshire, LS9 7TF UK; The Central Area of North Wales NHS Trust, Glan Clwyd Hospital, Sarn Lane, Rhyl, LL15 5UJ UK; Centre for Statistics in Medicine, Nuffield Department of Orthopaedics, Rheumatology and Musculoskeletal Sciences, University of Oxford, Windmill Road, Oxford, OX3 7LD UK

**Keywords:** haemorrhoids, stapled haemorrhoidopexy, excisional haemorrhoidectomy, haemorrhoid artery ligation, randomised controlled trials, discrete choice experiment, health economics, anorectal surgery, trial incentives

## Abstract

**Background:**

Current interventions for haemorrhoidal disease include traditional haemorrhoidectomy (TH) and stapled haemorrhoidopexy (SH) surgery. However, uncertainty remains as to how they compare from a clinical, quality of life (QoL) and economic perspective. The study is therefore designed to determine whether SH is more effective and more cost-effective, compared with TH.

**Methods/Design:**

**eTHoS (e**ither **T**raditional **H**aemorrhoidectomy **o**r **S**tapled Haemorrhoidopexy for Haemorrhoidal Disease) is a pragmatic, multicentre, randomised controlled trial. Currently, 29 secondary care centres are open to recruitment. Patients, aged 18 year or older, with circumferential haemorrhoids grade II to IV, are eligible to take part. The primary clinical and economic outcomes are QoL profile (area under the curve derived from the EuroQol Group’s 5 Dimension Health Status Questionnaire (EQ-5D) at all assessment points) and incremental cost per quality adjusted life year (QALY) based on the responses to the EQ-5D at 24 months. The secondary outcomes include a comparison of the SF-36 scores, pain and symptoms sub-domains, disease recurrence, complication rates and direct and indirect costs to the National Health Service (NHS). A sample size of n =338 per group has been calculated to provide 90% power to detect a difference in the mean area under the curve (AUC) of 0.25 standard deviations derived from EQ-5D score measurements, with a two-sided significance level of 5%. Allowing for non-response, 400 participants will be randomised per group. Randomisation will utilise a minimisation algorithm that incorporates centre, grade of haemorrhoidal disease, baseline EQ-5D score and gender. Blinding of participants and outcome assessors is not attempted.

**Discussion:**

This is one of the largest trials of its kind. In the United Kingdom alone, 29,000 operations for haemorrhoidal disease are done annually. The trial is therefore designed to give robust evidence on which clinicians and health service managers can base management decisions and, more importantly, patients can make informed choices.

**Trial registration:**

Current Controlled Trials ISRCTN80061723 (assigned 8 March 2010).

**Electronic supplementary material:**

The online version of this article (doi:10.1186/1745-6215-15-439) contains supplementary material, which is available to authorized users.

## Background

### The burden of the problem

Haemorrhoids are common in all age groups from mid-teens onwards. In 2006 and 2007, approximately 25,000 haemorrhoidal procedures were performed in England as hospital day-case or inpatient admissions, resulting in significant calls on health service resources [1]. The treatment of haemorrhoidal disease is directed at relieving its related symptoms. Traditional surgical haemorrhoidectomy (TH) involves excision of the haemorrhoidal cushions and is generally advocated for symptomatic haemorrhoids of grade III or IV. This traditional approach, whilst effective, is however associated with severe pain.

Improved understanding of the pathogenesis of haemorrhoids [2], increasing belief in the importance of preserving the anal cushions and greater awareness of the complications associated with excisional haemorrhoidectomy led to the invention of newer surgical procedures including stapled haemorrhoidopexy.

Stapled haemorrhoidopexy (SH) was conceived over 15 years ago and was first described by Longo [Longo A: Treatment of haemorrhoidal disease by reduction of mucosa and haemorrhoidal prolapse with a circular suturing device: a new procedure, unpublished]. Its potential advantages over traditional surgery include a reduction of operating time, hospital stay, time to return to work and postoperative pain [3]. These features would seem to make it attractive to patients and healthcare providers. Nevertheless, uncertainties around complication rates, recurrence of symptoms and costs preclude its widespread use across the National Health Service (NHS).

### The decision to evaluate clinical and cost-effectiveness of the two surgical treatments for haemorrhoids (stapled haemorrhoidopexy and traditional haemorrhoidectomy)

There have been multiple randomised controlled trials (RCTs) comparing SH with TH. These RCTs have been analysed in two systematic reviews and a Health Technology Assessment (HTA) monograph [4–6]. The HTA included a review of the clinical effectiveness data from 27 RCTs (n =2,279; 1,137 SH; 1,142 TH). When comparing SH with TH, the authors revealed equivalent complication and pain rates at day 21. However, SH patients had less pain in the immediate post-operative period compared with TH. Over the longer term, there was a significantly increased rate of residual prolapse requiring re-intervention with SH; however, there was no evidence of a difference in the number of patients experiencing pain or bleeding between SH and TH. The economic evaluations of the two interventions reported in the HTA monograph found that TH dominated SH, but it should be noted that TH and SH had very similar costs and Quality of Adjusted Life Years (QALYs). The additional cost of the stapling instrument was largely, but not completely, offset by savings in operating time and hospital stay. In terms of QALYs, the improvements in quality of life due to lower pain levels in the early post-operative period with SH were offset by losses in quality of life as a result of the higher rate of symptoms over the follow-up period. SH thus appears to be associated with less pain in the immediate postoperative period, but a higher rate of recurrence in the longer term and increased need for further surgery. These findings are based on data from small trials, all with methodological flaws, and providing limited data on quality of life (or with respect to an economic interpretation, health state utilities) in the early postoperative period. The study by Thaha and colleagues reported similar findings [7]. There are, however, a number of potential limiting factors in the applicability of this study. First, the SF-36 data used to measure quality of life did not rule out substantial differences, which only a larger trial would be able to detect. Second, the stapling gun has subsequently undergone refinement (recruitment was completed in 2002). Third, the trial was conducted prior to the stapling technique being well-established in the UK health care system.

Whilst there is a reasonable volume of work on grade III and IV haemorrhoids, there is a paucity of clinical and economic data regarding SH or TH for grade II haemorrhoids. Our group has conducted a RCT comparing rubber band ligation (RBL) with SH for grade II haemorrhoids using both clinical and economic outcomes [8]. This showed a superior clinical effect of SH compared to RBL in terms of recurrence of haemorrhoid symptoms. However from a health economic standpoint, SH, when compared with RBL could not be justified, even with a two-year follow-up. The trend over a longer period, however, suggested that the greater failure rate for RBL may eventually reach a level that justified the increased cost of SH. However, a larger trial with longer term follow-up is needed to confirm this.

This small trial used similar outcome measures to those being used in eTHoS and had a high return rate over a median follow-up period of 36 months. Internal reproducibility of the symptom score (the Haemorrhoid Symptom Score) was also validated in this trial by re-administration of questionnaires after an appropriate wash-out period [9]. This symptom score measures the presence, frequency and severity of key haemorrhoidal symptoms (prolapse, pain, bleeding, pruritis, seepage and incontinence for flatus or faeces). These symptoms are scored from 0 to 4 in each domain (except for pain, which scores from 0 to 2). The Cleveland incontinence score [10] is a standard measure of the degree of disturbance to life caused by incontinence. While it is evident that many patients with haemorrhoids have mild disturbance mainly related to flatus, the main utility is in detecting any problems related to sphincter injury as a result of surgery.

There is, therefore, a need for an adequately powered, high quality, multicentre RCT comparing the clinical and cost-effectiveness of SH compared with TH. Patient reported health status will be observed over the trial period as well as symptoms related to haemorrhoids, general health and complications from either procedure.

The aim of eTHoS is to assess whether SH is more effective and cost-effective compared with traditional excisional haemorrhoidectomy (TH) for people with haemorrhoids (grade II, III and IV).

**The primary objective** is to compare patient reported overall health related quality of life (measured using the EQ-5D) over a period of 24 months.

**The secondary objectives** are to compare sub-domains of health (SF-36 scores, pain and symptoms), disease recurrence, complication rates, and direct and indirect costs to the NHS, and cost-effectiveness (measured in terms of incremental cost per QALY, where QALYs are derived from responses to the EQ-5D).

## Methods/Design

eTHoS is a pragmatic, multicentre, superiority, parallel group trial comparing SH and TH.

### Trial recruitment and allocation

In order to run the study according to the protocol, each hospital centre participating in the eTHoS study will require at least two members of staff to occupy two key research roles. One research role is that of a (co-investigating) colorectal consultant; the other will be a local Recruitment Officer (RO) (for example, a nurse or junior doctor). In exceptional circumstances the colorectal consultant may perform both roles. At each centre there may be more than one colorectal consultant (co-investigator) who will be fully eTHoS-trained and actively screening potential patients (for eligibility) and subsequent recruitment onto the trial. At each centre, one of these consultants will assume the leading role of local lead colorectal surgeon for eTHoS. The RO will work with all of the named eTHoS study colorectal surgeons and, along with the lead, will administer the trial in accordance with the protocol. A list of participating sites can be obtained from the Chief Investigator, Professor Angus Watson (angus.watson@nhs.net).

#### People considered for trial entry

Inclusion criteria include the following:

1. patients with circumferential haemorrhoids grade ii, grade iii and iv,
2. patients aged 18 years or older and
3. written informed consent.

Exclusion criteria include the following:

1. previous surgery for haemorrhoids (traditional or stapled) (except rubber band ligation (rbl) or haemorrhoidal artery ligation operation (halo));
2. previous surgical treatment for anal sphincter injury repair, or symptomatic incontinence and peri-anal sepsis;
3. known inflammatory bowel disease;
4. malignant gastrointestinal disease, within the last five years;
5. medically unfit for surgery or for completion of the trial; or
6. pregnant women.

#### Recruitment and administration of follow-up procedure for eTHoS

Participating surgeons from each collaborating colorectal surgical unit will identify patients referred to the hospital for surgical treatment. Those patients meeting the eligibility criteria will be invited to enter the trial. Patients who accept the invitation to join the trial will be randomly assigned to be treated by either SH or TH. Outcome assessment will be at 1 week, 3 weeks and 6 weeks after surgery and 12 months, 24 months and (subject to securing further funding) 60 months after randomisation. If response to longer term follow-up (1 year or later) is lower than anticipated, approaches to address this will be considered, and research ethics committee (REC) approval will be sought if appropriate.

#### Recruitment procedure

Local procedures at the participating hospitals are different and the timing and mode of approach to patients and the consent process will vary to accommodate both the variability at the sites and the needs of the patients.

Eligible patients will be identified in the clinic setting by the colorectal surgeon or a suitably qualified trained member of the local clinical team and noted in an eTHoS log book. The colorectal surgeon will inform the patient during this initial consultation about the different treatments available for their condition as well as giving information about the eTHoS study. As is normal clinical practice, the colorectal surgeon will explain the risks and benefits of all the treatment options.

Informed consent will be obtained from all participants. The colorectal surgeon, or local trained clinical team member, will give each potential participant the Patient Information Sheet. This explains the rationale behind the eTHoS study, as well as what taking part encompasses.The colorectal surgeon, or locally trained clinical team member, will then be on hand to answer any questions/discuss the study with the potential participant during/immediately after this consultation appointment or at home. Patients will be encouraged to take home and re-read in detail the Patient Information Sheet (already given) during this time. If the patient agrees to be contacted at home, he/she may receive a telephone call from a local study team member to discuss the trial. Patients who decide to participate after telephone counselling can either send their completed documents (consent form and baseline questionnaire) through the post to the local study team at their treating hospital or bring it with them if/when they are returning to hospital for pre-op assessment or at the time of the operation.

Patients who are able to make a decision to join the study whilst they are at the clinic will be provided with the eTHoS participant baseline questionnaire that comprises the EQ-5D, SF-36, Cleveland Incontinence Score and Haemorrhoids Symptom Score. Contact details of both the local and central team are provided on the Patient Information Sheet. Patients who require more time to consider participation in the study will be encouraged to contact either the local or central team if they have any queries for which they would like clarification before they return to hospital. The potential participant will then be re-approached by a local clinical team member prior to surgery. If a patient does not return for pre-assessment (for example, if they live remotely or due to local site procedures), then the patient can return their signed consent form by post to their recruiting site. The form will be counter-signed on receipt by the local clinical team member. The patient will be advised to contact the site staff by telephone for further clarification or information if needed.

These arrangements will be individualised for each centre. Following full written consent and baseline data completion, patients will be randomised, as near to their surgery as possible, to one of the two study groups in equal proportion using the randomisation application at the trial office. Patients who return their signed consent forms by post will then complete the baseline questionnaire prior to surgery, to enable randomisation to take place.

The outcome of the recruitment consultation(s) with each potential eTHoS participant will be fully documented in an eTHoS log book. For those who consent to participate, a copy of their signed consent form will be filed in the patient’s hospital record. In addition, a copy will be given to the participant, a copy will be held in the investigator’s site file and the original will be retained by the trial office.

For those patients who do not consent to participate, an ‘Ineligible/Declined’ form will be completed by a local clinical team member, detailing non-personal data, including the reason(s) for the participant declining, or the ineligibility criterion. These data will be recorded on the study database.

#### Follow-up procedure

The eTHoS patient follow-up will consist of a visit to the hospital, approximately 6 weeks after surgery (range allowed 4 to 8 weeks), for a clinical consultation and assessment. At randomisation, both the participant and the surgeon will be aware of the treatment randomisation group. Data collected at all participant visits (including the initial consultation/eligibility visit) will be recorded in the first instance on paper case report forms (CRFs) then entered onto the trial database via a secure web portal.

The trial office will coordinate follow-up and data collection in collaboration with the UK centres. The study web portal will be the fulcrum of all trial documentation and facilitate communication between study personnel. The surgical form will be completed by the operating surgeon. All participant-reported outcomes (PROs) (apart from baseline) will be collected by postal questionnaires administered from the trial office.

#### Additional clinic or hospital visits

Data on any additional hospital visits will be recorded on the CRF completed when participants return for the 6-week clinical follow-up or in the patient reported outcomes.

#### Participant withdrawal

Participants will remain on the trial unless they choose to withdraw consent or if the principal investigator (PI), chief investigator (CI) or trial office feel it is no longer appropriate for the participant to continue (that is, participant becomes unable to complete the trial documentation). The reason for the participant being withdrawn from the trial will be recorded on the ‘withdrawal/change of status’ form, and if the participant is still willing to complete follow up questionnaires and/or to have relevant outcome data collected from NHS records, then the follow-up process will continue.

#### Training

Training and support will be given in a standardised format to both the colorectal surgeons and the ROs. Training, by a member of the study team, will focus on the eTHoS trial flowchart and the protocol. Training in physical baseline and follow-up measurements will also be given to the ROs if required. The colorectal surgeons and the ROs will use standard study instruction manuals and documentation, which will be provided by the study office, for reference and support throughout. The study office will also be the first point of contact for the colorectal surgeons and the ROs in case of problems, concerns, adverse effects or the need for advice. RO training days will also be held in a variety of UK locations.

#### Specific eTHoS roles and responsibilities

It is envisaged that the duties of the principal local investigator, co-investigating colorectal surgeons and the ROs will be managed among them according to capacity and in accordance with the eTHoS protocol.

### Randomisation and allocation

Participants will be randomised to one of the two study groups in equal proportion using a randomisation application in the trial office. This randomisation application will be available 24 hours a day, 7 days a week and has both an Interactive Voice Response (IVR) telephone and web-based interface. Randomisation will take place as near to the time of surgery as possible using minimisation.

The minimisation algorithm [11] will use centre, grade of haemorrhoidal disease (II, III or IV), baseline EQ-5D score and gender.

### Trial interventions

Eligible and consented participants will be placed on the appropriate waiting list by the treating colorectal surgeon or his/her designated team member. Participants will receive the allocated intervention, either SH or TH. Each centre’s participating surgeons must have undergone appropriate recognised training for both stapled and traditional haemorrhoid surgery. Ideally, this will have included attendance at a ‘master class’. Surgery can be performed by surgeons in training, either independently, if signed off by their supervising consultant, or under the direct supervision of their consultant. Pre- and post-operative care is to follow the respective surgeon’s and centres standard policies.

Baseline data and follow-up measurements are recorded throughout the study on the eTHoS Case Report Forms (CRF).

**Figure 1:**
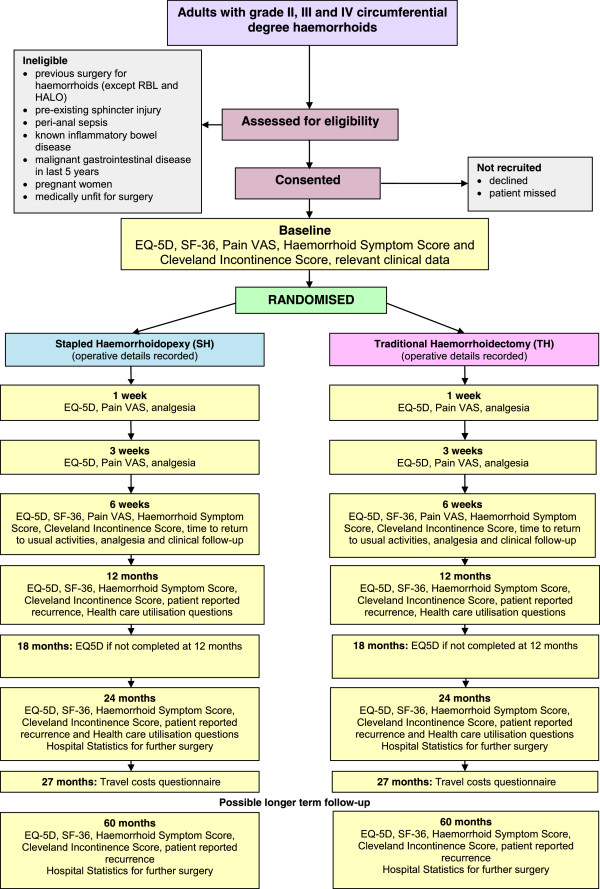
**Flow diagram of the eTHoS trial.** EQ-5D, European Quality of Life 3 Dimensions; VAS, visual analogue scale; SF36, Short Form (36) Health Survey.

See Figure 1 for an overview of the trial.

### Stapled haemorrhoidopexy

The patient will undergo stapled haemorrhoidopexy (SH). Each centre must house experienced surgeons who have undergone appropriate surgical training to perform SH.

• SH aims to correct haemorrhoidal prolapse by excising a ring or ‘donut’ of tissue above the haemorrhoidal cushions with immediate re-anastomosis of the mucosa using staples. A secondary effect may be to reduce blood flow and therefore congestion. Fibrosis develops at the staple line maintaining the haemorrhoids in their new position. The main stapling gun in use in the United Kingdom is the PPH03 (Ethicon Endo-Surgery (Europe) GmbH, part of the Johnson & Johnson family of Companies, Norderstedt, Germany), which is used by the majority of colon and rectum surgeons. Covidien (Covidien, New Haven, Connecticut, USA) have a dedicated stapling instrument for haemorrhoidal surgery that is similar in design to the stapler provided by Ethicon Endo-Surgery. Chex Healthcare (Chex Healthcare, Frankenman International Ltd., Kowloon, Hong Kong) is newer to this market and has produced a stapler that is very similar to the one made by Johnson and Johnson. There are some key differences: it is around 40% cheaper and has a design that may make it easier to use in male patients. SH is conducted using a stapling gun. Reflecting the pragmatic nature of the trial, surgeons will be able to use the gun they would normally use in practice.

### Traditional excisional haemorrhoidectomy

There are two main excisional procedures currently carried out: open (Milligan and Morgan) and closed (Ferguson). Both have the intention of excising the haemorrhoidal cushions and are traditionally associated with severe postoperative pain. The apparent efficacy of the procedures may be, in part, due to reluctance of patients to seek further treatment in the light of previous experience. Participating surgeons are required to have undergone appropriate surgical training and be competent to perform traditional excisional haemorrhoidectomy (TH).

### Subsequent arrangements

#### Notification of GPs

General practitioners (GP) will be notified by letter, which includes a GP eTHoS information sheet, that their patient has been randomised to the eTHoS study. GPs are asked to phone the Study Office if the participant moves, becomes too ill to continue with the study, dies, or any other notifiable event/possible adverse event occurs. Alternatively, staff at the study office may contact the GP.

#### Flagging on central medical databases

Consent will be sought from all participants recruited to the RCT to be flagged for notification of haemorrhoidal recurrence. To evaluate long term safety, the participants will be flagged for further haemorrhoidal surgery through Hospital Episode Statistics (HES) in England and Patient Episode Database Wales (PEDW) in Wales and the Information Services Division (ISD) data in Scotland, when all participants have reached 12 and 60 months.

#### Safety

We will report serious adverse events in accordance with the guidance from the National Research Ethics Service (NRES), which is a subdivision of the National Patient Safety Agency.

#### Possible expected occurrences

In this study a number of potential occurrences are expected. Possible (expected) intraoperative occurrences associated with the intervention include anaesthetic related problems, intra-operative instrument failure, damage to adjacent organs and bleeding. Possible (expected) occurrences associated with either type of surgery occurring at any time during the trial include haemorrhage, requirement for blood transfusion, anal stenosis, anal fissure, pain, urinary retention, residual anal skin tags, anal fistula, prolapse, difficult defecation, faecal urgency, wound discharge, pelvic sepsis, systemic complications and pruritis.

Details of any of the occurrences listed above will be recorded on the case report forms and participant completed questionnaires and reported to the data monitoring committee (DMC).

#### Procedure for reporting untoward and related serious adverse events in this study

A serious adverse event (SAE) in the eTHoS trial is defined as when one of the following events occurs to a research participant:

1. related (resulted from administration of any of the research procedures) and
2. expected or unexpected (that is, the type of event that is not listed above as an expected serious occurrence) that causes death, is life threatening, requires hospitalisation, results in significant incapacity/disability or is otherwise considered medically significant by the investigators.

All SAEs will be recorded on the Serious Adverse Event Report form. In addition, SAE forms will record all deaths due to any cause during the course of the study.

#### Reporting responsibilities of the chief investigator

When the web-based Serious Adverse Event form is completed detailing any possible related and unexpected SAEs, the chief investigator (CI) will be notified automatically. If, in the opinion of the local investigator and CI, the event is confirmed as being related and unexpected, the CI will submit a report to the main REC and the study sponsors within 15 days of the CI becoming aware of it or within 7 days if it is a death (related to the study).

### Measures of outcome

The study has a patient-centred and an economic primary outcome, and multiple secondary patient-reported, clinical and economic outcomes.

#### Primary

The patient-centred outcome is the quality of life profile over the follow-up period (area under the curve derived from EQ-5D measurements at baseline, 1 week, 3 weeks, 6 weeks, 12 months, EQ-5D at 18 months only if not completed at 12 months, 24 months and (subject to securing further funding) 60 months.

The trial economic outcome is the incremental cost per quality adjusted life year (QALY) gained with QALYs based on the responses to the EQ-5D at 24 months.

The economic model outcome is the incremental cost per QALY over the lifetime of the participant.

#### Secondary

The patient-reported outcomes include the following:

1. generic health profile measured by SF-36 and EQ-5D,
2. visual analogue scale (VAS) pain score,
3. Cleveland incontinence score,
4. haemorrhoid symptom score,
5. post-operative analgesia consumption,
6. recurrence of haemorrhoids, and
7. tenesmus.

Clinical outcomes are perioperative and postoperative complications including the following:

1. haemorrhage,
2. requirement for blood transfusion,
3. anal stenosis,
4. anal fissure,
5. urinary retention (which requires catheterisation),
6. residual anal skin tags,
7. difficult defecation,
8. wound discharge,
9. pelvic sepsis, and
10. pruritus.

Economic outcomes will be the costs based on resource use data and include the following:

- Costs to the NHS and patients at two years
- time to recovery
- length of hospital stay
- use of health services for haemorrhoid related events or treatments
- patient costs (treatments, travel to health services, sick leave)
- need for alternative management for haemorrhoids (for example, surgery, drugs)
- other use of health services
- visits to GP

ii. visits to practice nurse

iii. visits to colorectal surgeon

2. Estimated lifetime cost to NHS and patient
3. QALYs estimated from the EQ-5D at 24 months
4. QALYs estimated over the patient’s lifetime
5. Cost-effectiveness analysis (incremental cost per case of stapled haemorrhoidopexy and traditional haemorrhoidectomy excision avoided).

### Data collection and processing

Follow-up will consist of clinical follow-up at 6 weeks and postal questionnaires at 1 week, 3 weeks, 6 weeks, 12 months (and 18 months if EQ-5D not completed at 12 months), 24 months and (subject to securing further funding) 60 months, with the main outcome assessment planned once the 24-month (from the date of randomisation) follow-up is complete.

#### Measuring outcomes

In this study the colorectal surgeon and the participant will know which intervention the participant has received. Clinical outcomes will be collected by the ROs and the colorectal surgeons. CRFs can be obtained from the Trial Office by contacting ethos@abdn.ac.uk. Table 1 shows the eTHoS schedule for physical assessment/data collection.

**Table 1:** **eTHoS schedule for physical assessment/data collection**

	Baseline	Surgical form	1 week	3 weeks	6 weeks	12 months	18 months	24 months	27 months	60 months
Clinical status CRF or data	○				○					
Surgical details		○								
Patient preference	○									
6 weeks clinical follow-up					○					
EQ-5D	○		●∞	●∞	●∞	●∞	●∞	●∞		●∞
SF-36	○				●∞	●∞		●∞		●∞
Pain VAS			●∞	●∞	●∞					
Haemorrhoid symptom score	○				●∞	●∞		●∞		●∞
Cleveland incontinence score	○				●∞	●∞		●∞		●∞
Health care utilisation questions						●∞		●∞		●∞
Travel costs questionnaire									●∞	
Recurrence						●∞x		●∞		●∞x
Analgesia question			●∞	●∞	●∞					
DCE								∞ ●		
Hospital statistics for further surgery						●∞x				●∞x

○ Clinic; ● Postal; **x** HES, PEDW and ISD; ∞Web based. CRF, case report form; DCE, discrete choice experiment; EQ-5D, EuroQol Group’s 5 Dimension Health Status Questionnaire; SF-36, Short Form Health Survey 36 Question form; VAS, visual analogue score.

#### Patient reported outcomes

At baseline, (recruitment) participants will complete the patient reported outcome (PRO) questionnaires. In addition at 1 week, 3 weeks, 6 weeks, 12 months (EQ-5D only at 18 months if not completed at 12 months), 24 months and (subject to securing further funding) 60 months, participants will complete the eTHoS PRO questionnaires. These will be distributed by post and completed by the participant. Participants will be given the option to complete the 1-week, 3-week, 6-week, 12-month (EQ-5D only at 18 months if not completed at 12 months), 2-year and 5-year participant reported outcome questionnaires on a secure participant portal within the eTHoS website. Participants will be provided with a log-in to access the portal. In the event that these postal questionnaires are not returned, participants will be telephoned to obtain the missing data for the 1- and 3-week questionnaires. A postal reminder will be sent if there is no response to the 6-weeks, 12-month, 24-month and (subject to securing further funding) 60-month questionnaires. If they are not returned, a second reminder will be sent. If questionnaires are returned but not adequately completed, (that is, key outcome data are missing), either a member of the study office team or the RO, as appropriate, will telephone the participant and obtain the missing questionnaire data as required.

#### Healthcare utilisation

NHS costs for health services use in both secondary and primary care by the UK trial participants will be collected.

At 12, 24 and (subject to securing further funding) 60 months after randomisation, participants will provide information about their use of health services (via the health care utilisation questions within the eTHoS patient reported outcome instrument. A postal questionnaire survey of all participants will be used to ascribe costs to typical episodes of health service use (the Participant Travel Cost Questionnaire) sent approximately 27 months after randomisation. The underlying aim is to keep economic data collection as parsimonious as possible to minimise the burden on the participants and the effect on response rates.

#### Patient preference (baseline and discrete choice experiment)

Burch and colleagues [6] found that the two treatments differed in terms of short-term outcomes (earlier return to usual activities, pain) and differed in terms of the risk of recurrence. Quality of life measurement (and QALYs based upon them) may not fully represent patients’ preferences for treatments and their associated outcomes. Given this, global patient preference will be elicited at baseline using a single 5-point Likert scale response to a hypothetical example. Furthermore, a discrete choice experiment (DCE) to allow an in-depth elicitation of the individual strength of preference for the different treatments during the follow-up period will be conducted. The choice of attributes will relate to the trial outcome measures and reflect advice from members of the trial team, as well as evidence from the appropriate literature. One further attribute of the DCE will be patient cost, which will allow willingness to pay (WTP) to be estimated. In particular, willingness to pay for specific attributes of treatment will be assessed. Estimating willingness to pay from the DCE will enable these estimates to be combined into the broader economic evaluation. The DCE will also include an additional surgical intervention, haemorrhoidal artery ligation (HAL) to take into account all the treatment options available to patients with haemorrhoids.

The DCE will describe the intervention in terms of a number of characteristics (attributes), for example, time in short-term pain and risk of recurrence. The extent to which an individual values an intervention will depend upon the levels of these attributes [12, 13]. The DCE technique involves presenting choices to individuals that imply a trade-off in terms of the levels of the attributes. To define the attributes and levels for the DCE, a literature review will be conducted as well as taking expert advice on potential attributes from members of the research team. Once the attributes and levels are defined, experimental design techniques will be used to reduce the number of possible choice sets to a manageable size, whilst still being able to estimate utility scores. In addition to the choices derived from the experimental design, two choice sets will be added to test the internal consistency of responses. These will be dominant (better) choices for one option and respondents would be expected to choose them.

The questionnaire will be piloted amongst a small sample (members of the research group and Health Services Research Unit, Aberdeen) to refine all practical aspects of the survey and to ensure that respondents are making trade-offs between the attributes. Once the pilot is complete and the questionnaire refined, a proportion of trial participants (n =100) will be sent the DCE questionnaire. The questionnaire will be sent to an online survey panel of non-trial participants. Generalised linear (for example, logistic) regression models will be used to analyse the response data. The decision on which statistical model to use to analyse the data is an empirical one and will depend to a certain extent on the final data collected.

#### Data processing

Clinical data will be collected at the individual hospital centres using, where necessary, hospital-based records and hardcopy CRF forms. These clinical data will then be input into the eTHoS database by local researchers using an electronic web-based data capture system (in addition, relevant clinical data will be collected from routine data sources (HES, PEDW & ISD)). Extensive range and consistency checks will enhance the quality of the data. Staff in the Study Office will provide periodic data queries to local research staff to ensure that the data are as complete and accurate as possible.

### Analysis plans

The full details of the statistical analysis plan (SAP) can be obtained from the Trial Office by contacting ethos@abdn.ac.uk.

#### Ground rules for the statistical analysis

Study analyses will follow the SAP agreed in advance by the Trial Steering Committee. The main statistical analyses will be based on all participants as randomised, irrespective of subsequent compliance with the treatment allocation.

The primary outcome, area under the curve (measured by EQ-5D), will be generated for each participant using the trapezoidal rule. Missing EQ-5D data will be estimated using a multiple imputation approach which makes use of partial outcome data [14]. Sensitivity analyses will be conducted to assess the robustness of the treatment effect estimate to these approaches. The primary outcome measure will be analysed using linear regression with adjustment for the minimisation variables. Secondary outcomes will be analysed using generalised linear models with adjustment for minimisation and baseline variables as appropriate. Statistical significance will be at the two-sided 5% level with corresponding confidence intervals derived. Subgroup analyses will explore the possible treatment effect modification of clinically important factors (grade and gender), through the use of treatment by factor interaction, all using a stricter two-sided 1% level of statistical significance.

An independent Data Monitoring Committee (DMC) will meet early in the course of the trial to agree to its terms of reference and will review confidential interim analyses of accumulating data.

#### Timing and frequency of analyses

A single principal analysis is anticipated once the final participant has reached the 24 months time point. The DMC will determine the frequency of confidential interim analyses. The potential for analysing longer-term follow-up data (post 24 months) will be assessed once the principal analysis has been carried out.

#### Planned subgroup analyses

Subgroup analyses are planned to investigate the influence of haemorrhoidal grade and gender.

#### Economic analysis

##### Costs of management of haemorrhoids for eTHoS participants

Participant costs will comprise three main elements: self purchased health care, travel costs for making return visit(s) to NHS health care, and time costs of travelling and attending NHS health care.

Self-purchased health care is likely to include items such as prescription costs and over-the-counter medications. Information about these will be collected through the healthcare utilisation questions.

Estimation of travel costs requires information from participants about the number of visits to, for example, their GP or consultant (estimated from the healthcare utilisation questions) and the unit cost of making a return journey to each type of healthcare provider (from the Participant Unit Cost Questionnaire).

The cost of participant time will be estimated in a similar manner. The participant will be asked, in the Participant Unit Cost Questionnaire, how long they spent travelling to and attending their last visit to each type of healthcare provider. Participants will also be asked what activity they would have been undertaking (for example, paid work, leisure, or housework) had they not attended the healthcare provider. These data will be presented in their natural units (for example, hours) and also as cost estimates using standard economic conventions (for example, the Department of Transport estimates for the value of leisure time). These unit time costs, measured in terms of their natural and monetary terms, will then be combined with estimates of the number of healthcare contacts derived from the healthcare utilisation questions.

##### Costs of intervention

The costs of the surgical interventions will be recorded on a per patient basis. The resources used to provide surgery will be calculated by consulting with relevant staff (surgeons, theatre nurses, business managers) and members of the trial team to elicit information on the following:

1. reusable equipment,
2. frequency of use of that equipment,
3. consumables used during surgery,
4. staff mix of the surgical team and
5. overheads costs for specific time periods.

In addition to this, the operative details will be collected on the CRFs and will provide estimates of the grade of operator, assistant and anesthetist, as well as relevant procedure times.

Unit costs for these resources will be based on nationally available data and study-specific estimates. Longer term estimates of resource use and cost will be derived from trial estimates and the literature.

Length of stay information will be elicited for each patient through the case report forms by collecting the date of admission and discharge. Unit costs for each level of care will be initially obtained from the Scottish Health Service Costs (SHSC) [15] for the primary analysis and NHS National Reference Costs in a secondary analysis. These sources will not have a cost per day for all hospital services; therefore some calculations will be needed to determine the ‘cost per day’ for each level of care.

##### Costs of subsequent care

The number of outpatient visits per patient for each relevant specialty will be obtained from the CRFs. Unit costs for outpatient visits will initially be obtained from the SHSC [15] for the primary analysis and National Reference Costs in the sensitivity analysis [16].

The number of general practice contacts, for example GP office or home visits or phone consultations, will be obtained from the Health Service Utilisation Questionnaire. Unit costs for GP visits will be obtained from the Personal Social Services Research Unit (PSSRU) unit costs of community care [16]. For each patient, the number of visits will be multiplied by the appropriate unit cost. These costs will be summed to produce a total cost per patient. When a cost for each patient has been estimated, a mean cost for each intervention group will be calculated.

Any reoperations or new surgical interventions will be identified from the CRFs and for the associated cost estimated using data from routine data sources [15, 16] or operation costs previously estimated for the study. Any duration of any relevant admissions during the follow-up period will be estimated from the CRFs and the associated cost estimated using the methods described above.

##### Cost effectiveness

As part of this study, an economic evaluation will be conducted. It will be based on both a modelling exercise and a ‘within trial’ analysis. Either an existing, or *de novo,* economic model will be used to assess the relative cost-effectiveness (assessed in terms of incremental cost per QALY) and net benefits of SH and TH. A model was developed as part of a recent HTA-funded project, and we have negotiated access to that model [6]. Our group has also developed a model to compare the cost-effectiveness of SH and RBL for grade II haemorrhoids. We will critique these models and use, or adapt them, to address our study question. If necessary we will use the lessons learnt from these models to develop a new model that better addresses the research question. The data from the trial will be the main source of data for the modelling but further data with which to model outcomes beyond a 24-month follow-up will be systematically derived from the literature and other existing data sources following guidance for best practice [17].

Data collection from the trial will focus on estimating the use of secondary and primary care resource use and on health state valuations obtained from EQ-5D. Resource use and patient costs will be obtained from participant-completed questionnaires at 12 and 24 months. Unit cost will be based on nationally available data and study-specific estimates. Longer term estimates of resource use and cost will be derived from trial estimates and a structured review of the literature. QALYs will be estimated from the responses to the EQ-5D valued using the UK population tariffs.

The results of the economic model will be supplemented by a within-trial analysis. This analysis will use the estimates of costs and QALYs estimated for each trial participant to calculate the incremental cost-effectiveness ratios for the 24-month follow-up, and where appropriate, the analysis will mirror the statistical analysis (for example, incremental costs and QALYs will be adjusted for the minimisation variables using regression techniques). To facilitate interpretation of the trial results, the within-trial economic analysis will also be presented in the form of a balance sheet where differences in terms of benefits and costs of the two trial interventions are presented in their natural or clinical units.

The perspective of the model and within-trial analyses will be the patient and the UK NHS. The results of the analyses will be presented as point estimates of mean incremental costs, effects and incremental cost per QALY. Sensitivity analysis will be applied to the model in order to assess robustness of the results to realistic variations in the levels of the underlying data and also alternative assumptions, for example, QALYs derived from the SF-36. This will be accomplished using probabilistic and deterministic sensitivity analyses to address parameter and other forms of uncertainty. Similarly, for the within-trial analysis, techniques such as bootstrapping will be used alongside deterministic sensitivity analyses to address uncertainty. In both the model and the within trial analyses the cost per QALY data will be presented in terms of cost-effectiveness acceptability curves (CEACs).

##### Discrete Choice Experiment analysis

The results of the DCE will be combined with the clinical outcomes estimated from the trial or model to provide an estimate of the mean WTP for each intervention considered for both the model based and the within trial analyses. Results will be presented as incremental net benefits (Net benefits = mean WTP - mean cost for each intervention). The intervention with the greatest net benefit would be considered the most efficient. For the model- and trial-based analyses, probabilistic, or stochastic (for the trial based analysis), along with deterministic sensitivity analyses, will be constructed.

### Sample size and feasibility

#### Sample size sought

A sample size of n =338 per group is required to provide 90% power to detect a difference in the mean area under the curve (AUC) of 0.25 standard deviations derived from EQ-5D score measurements, with a significance level of 5% (two-sided alpha). Good data on 24-months AUC for this instrument in this patient group is sparse, but a 0.25 effect size has often been shown to correspond to a worthwhile difference in quality of life measures. This would equate to a difference of 0.1 in the AUC (QALY) assuming a standard deviation of 0.4. Evidence-based strategies will be used to enhance questionnaire response rates in this highly motivated group of patients. Conservatively, to allow for 15% non-response in the outcome, it is proposed to randomise 400 subjects in each of the two groups. Such a sample size would provide 90% power to assess differences in the secondary outcome of recurrence between the two surgical techniques from around 10% to around 4%. This magnitude of difference is supported by a recent systematic review which showed a non-statistical trend higher recurrence in the SH group compared to TH group [4].

#### Recruitment rates

Previous experience of recruitment in NIHR/MRC surgery trials co-coordinated from the trial office, suggests that around 50% of those eligible will agree to be randomised. The recruitment period is anticipated to last 43 months (months 4 to 46 inclusive). Around 1,600 eligible patients are likely to have to be approached to randomise the required 800.

### Organisation

It is anticipated that there will be bi-annual PMG, six meetings of the TSC and five of the DMC. Two meetings are planned for collaborators (including the collaborating colorectal surgeons), the first timed to occur when all the centres have been identified and the second when results are available.

#### Local organisation in centres

##### Lead colorectal surgeon

Each collaborating centre will identify a lead colorectal surgeon (principal investigator (PI)) who will be the point of contact for that centre. The PI will take responsibility for ensuring that the outcome measures are taken consistently and in line with the standardised protocols developed for the study. Specifically, this person will do the following:

1. accept overall responsibility for the eTHoS study locally;
2. assist the eTHoS study office in establishing the study locally (for example agreement from clinical colleagues, helping the main study office to facilitate local trust approval, identify and appointing an RO and informing all relevant local staff about the study);
3. identify eligible patients;
4. explain the eTHoS study and take informed consent;
5. take overall lead responsibility for ensuring that the outcome measures are taken consistently and in line with the standardised protocols developed for the study;
6. take overall lead responsibility for all clinical aspects of the study locally (for example, if any particular concerns occur);
7. notify the eTHoS study office of any unexpected clinical events which might be related to study participation;
8. provide support and supervision for the local RO;
9. represent the centre at the collaborators’ meeting;
10. place patients who are randomised to SH or TH on the waiting list for surgery;
11. complete fully the appropriate eTHoS paperwork for patient participation; and
12. facilitate/supervise/participate in the upload of this hardcopy patient data to the web based system.

##### Recruitment officer (RO) at each centre

Each collaborating centre will appoint a RO to organise the day to day running of the study in that centre. The overall responsibilities of this person will be as follow:

1. work with the PI and other local colorectal surgeons in order to organise the day to day recruitment and follow-up of eTHoS participants of the study in that centre;
2. keep regular contact with the PI and other colorectal surgeons, notifying them of any problem or unexpected development;
3. maintain regular contact with the Study Office (including mailing of relevant material to the Study office);
4. keep local staff informed of progress in the study;
5. organise and supervise alternative recruiters in case of holiday or absence; and
6. represent the centre at the collaborators’ meeting if required.

The specific responsibilities of this person will be as follows:

1. Assist the PI and other local colorectal surgeons to keep a log of whether eligible participants are recruited or not (with reasons for non-participation)
2. Assist the PI and other colorectal surgeons in the distribution of the Patient Information Sheet and the collection and organisation of the patient consent forms
3. As appropriate organise follow-up to consultation appointments at 6 weeks after surgery with eTHoS participants
4. Ensure timely processing of consent and patient data (complete on-line baseline and follow-up clinical-data collection forms and enter into web application)
5. Undertake baseline measurements and follow-up measurements as appropriate and in accordance with eTHoS standard operating procedures
6. Support completion (as appropriate) of research questionnaires with the patients both face to face (at baseline) and when required during follow-up, including over the telephone as indicated from the eTHoS Study Office (that is, in the case of non return or significant missing data)
7. Act as a point of contact for the participants at all times and provide information about the trial, as necessary.

#### Study co-ordination in Aberdeen

##### The study office team

The Study Office is in the Centre for Healthcare Randomised Trials (CHaRT) based within the Health Services Research Unit, University of Aberdeen and provides day to day support for the clinical centres. The trial manager in CHaRT at Aberdeen will take responsibility for the day-to-day transaction of study activities. The data co-ordinator will provide clerical support to the trial, including organising all aspects of the postal questionnaires (mailing, tracking, and entering returned data using the study web data entry portal). The senior IT manager will oversee all IT aspects of the study, while the senior trials manager will provide mentoring and guidance to the trial manager and advice to the team on generic coordination issues. The programmer will create, maintain and update all applications programmes for the trial, including the randomisation application and all administrative and analysis databases. The trial statistician, under the supervision of a senior statistician, will be responsible for transacting all statistical elements of the study (including contributing to the pre-specified SAP and writing the statistical code that will implement this SAP, and producing progress reports for all the study committees (including the TSC and DMC). The economist, under the supervision of a senior economist, will take responsibility for all aspects of the economic evaluations integral to the study. The CHaRT quality assurance manager will ensure that CHaRT’s standard operating procedures for trials have been followed and properly documented, including observance of GCP throughout. At the centres, the recruitment coordinators will be responsible for all local processes involved in identifying, consenting and randomising the participants, along with facilitating the delivery of the intervention, under the supervision of the lead colorectal surgeon.

The eTHoS study office team will meet formally at least monthly during the course of the study to ensure smooth running and trouble-shooting. Finally, we intend to produce a yearly eTHoS Newsletter for participants and collaborators to inform everyone of progress and maintain enthusiasm.

#### The project management group

The study is supervised by its project management group (PMG). This consists of the grant holders and representatives from the Study Office. Observers may be invited to attend at the discretion of the PMG. The PMG will meet/teleconference every six months on average.

The research team has the expertise to cover the clinical and surgical aspects of the research. All the consultant surgeons involved have extensive surgical experience of stapled haemorrhoidopexy. Messrs Loudon (Cochrane review), Jayne (HTA systematic review) and Watson have experience in the design and conduct of RCTs involving SH. Messrs Loudon, Jayne, Maw and Brown have published extensively on SH. Messrs Watson, Loudon, Jayne and Brown are SH trainers.

#### The trial steering committee

The study is overseen by an independent trial steering committee. The other members are the grant holders. Observers or members of the host university (Aberdeen) and the funders (HTA) may also attend, as may other members of the PMG or members of other professional bodies at the invitation of the chair. Terms of reference for the TSC can be accessed upon request from the eTHoS study office.

#### Research governance, data protection and sponsorship

##### Research governance

The trial will be conducted according to the principles of good clinical practice provided by the MRC guidelines, the detail of which can be viewed at the following link: http://www.mrc.ac.uk/documents/pdf/good-clinical-practice-in-clinical-trials/ or in line with local implementation of research governance to at least the standard of the Aberdeen University policy on research governance, which can be viewed at the following link: http://www.abdn.ac.uk/staffnet/governance/research-governance-278.php.

##### Data protection

The trial will comply with the Data Protection Act 1998 and regular checks and monitoring are in place to ensure compliance. Data are stored securely in accordance with the Act and archived to a secure data storage facility. The senior IT manager (in collaboration with the CI) will manage access rights to the data set. Prospective new users must demonstrate compliance with legal, data protection and ethical guidelines before any data are released. We anticipate that anonymised trial data will be shared with other researchers to enable international prospective meta-analyses.

All data collected and stored within the study will comply with the Data Protection Act.

The Health Services Research Unit, University of Aberdeen Protecting Information Policy will be adhered to and can be accessed via this link: http://www.abdn.ac.uk/hsru/documents/Protecting_information_policy_v5_Dec13.pdf.

##### Sponsorship

NHS Highland and the University of Aberdeen are the co-sponsors for the trial.

#### Data and safety monitoring

##### Data monitoring committee

A separate and independent data monitoring committee (DMC) will be convened. A copy of the DMC charter can be obtained by contacting the Trial Office on ethos@abdn.ac.uk. It is anticipated that the members will meet once to agree terms of reference and on at least three further occasions to monitor accumulating data and oversee safety issues. This committee will be independent of the study organisers and the TSC. During the period of recruitment to the study, interim analyses will be supplied, in strict confidence to the DMC, together with any other analyses that the committee may request. This may include analyses of data from other comparable trials. In the light of these interim analyses, the DMC will advise the steering committee if, in its view, there are any ethical or safety issues that may necessitate modification to the protocol or closure of the trial.

The TSC, PMG, clinical collaborators and study office staff (except those who supply the confidential analyses) will remain ignorant of the interim results.

The frequency of interim analyses will depend on the judgement of the Chairman and other independent DMC members. We anticipate that there might be two interim analyses and one final analysis.

##### Safety concerns

Haemorrhoidal surgical treatment is a very common surgical procedure performed routinely by colorectal surgeons. However, as with all colorectal surgery, there are potential complications (see section on Safety), and these will be carefully monitored throughout the study.

In terms of general hazards of undertaking a large multi-centre RCT, all of (i) the safety of the participants, (ii) the scientific integrity of the study, and (iii) value for money for the public funder has been safeguarded by having the following: (a) a formal Clinical Trial Risk Assessment carried out by the University of Aberdeen and NHS Highland in their role as sponsors, (b) an excellent track record of the applicants in delivering successful multi-centre trials, (c) the support of a dedicated UKCRC registered Trials Unit (CHaRT at University of Aberdeen) and (d) excellent governance of the trial conduct by an experienced internationally recognised TSC and DMC.

Collaborators and participants may contact the chairman of the TSC through the study office about any concerns they may have about the study. If concerns arise about procedures, participants or clinical or research staff (including risks to staff), then these will be relayed to the Chairman of the DMC.

#### Ethical approval

The North of Scotland Research Ethics Committees reviewed and approved this study on 18^th^ June 2010 (REC reference number, 10/S0802/17).

Important amendments to this protocol will be communicated via email or letter as appropriate to the relevant parties (for example, sponsors, REC, participants, collaborators, trial registry).

## Discussion

The eTHoS study is the largest ever, randomised controlled trial on benign ano-rectal disease [18]. The trial is designed to answer an important question regarding the clinical and economic effectiveness of two surgical operations for haemorrhoids. Haemorrhoidal disease is very common in the developed world, and around 29,000 operations are performed in the UK on an annual basis [6].

Both operations have been available for over 15 years, but there has never been a rigorously conducted, large-scale, multicentre evaluation comparing both interventions. This is frequently the case when surgical technology is introduced into the market. Devices and operations penetrate practice through insidious adoption without due diligence being performed. This trial is designed to assess whether the newer operation, stapled haemorrhoidopexy, is more or less effective and cost-effective than the traditional surgery of excisional haemorrhoidectomy.

Surgical interventions are being continuously designed and the eTHoS trial is being run concurrently with other multicentre RCTs on haemorrhoid disease. The HubBLe trial [19] (of which the CI is a co-applicant) is comparing haemorrhoidal artery ligation with rubber band ligation for grade II and III haemorrhoids, whilst the LIGALONGO trial [20] being conducted in France is randomising between stapled haemorrhoidopexy and Doppler-guided arterial ligation with mucopexy [21]. These three multicentre RCTs are concurrently comparing the clinically relevant surgical interventions for haemorrhoids at the same time.

Whilst the running of parallel evaluations is exciting, it also presents a challenge to recruitment, as several recruiting centres are randomising patients to both trials within the UK. The adoption of HAL within surgical practice, prior to rigorous evaluation, has also had an impact on recruitment, as surgeons are keen to adopt and trial new surgical technologies. As such, the trial has been slower to recruit than originally expected. The recruitment period was therefore extended by 15 months to accommodate the short fall. At the time of publication, over 750 participants have been randomised in the trial.

The fundamental trial outcomes remain unchanged; however, there have been a number of amendments to the conduct of the trial, since its inception. The majority of these have centred on patient retention and follow-up. It is essential to the conduct of the trial that the questionnaire response rate is high. We introduced several initiatives during the recruitment period including a within-trial study, called to incentivise or not to incentivise. We aimed to test the null hypothesis that incentivisation would make no difference to questionnaire return rate. Participants were randomised to receive a five pound Sterling voucher with their 12- and 24-month follow-up questionnaires on the assumption that monetary incentivisation had worked in other trials [22].

Potential eTHoS trial participants were also invited to be involved in a further methodology project. This study sought to prospectively measure potential trial participant’s readiness to participate in a RCT. It is being conducted across a range of different clinical conditions and trials and seeks to evaluate how well informed patients feel before making a choice to participate in a trial.

Quality of life assessments may not completely represent patients’ preferences for treatment and their associated outcomes. We therefore planned a discrete choice experiment (DCE) to explore, using a number of procedure attributes, what factors are important for potential patients, when they are offered a variety of therapy options. The choices include potential complications (for example, pain, bleeding, and recurrence) and how much money a participant would be willing to pay for a procedure. We included the three most currently performed surgeries, excisional haemorrhoid surgery, stapled haemorrhoid surgery and haemorrhoidal artery ligation.

eTHoS is a large surgical trial that will enable the effectiveness and cost-effectiveness of SH and TH to be assessed. Taken together with HubBLe and LIGALONGO, the three multicentre trials will help inform patients, clinicians and health commissioners about the clinical utility and outcomes of competing treatments for haemorrhoids. It is hoped that in the future it will be possible to stratify treatment according to patient characteristics based on more robust evidence.

### Availability of the protocol

The full protocol can be obtained from the funder on the following link

http://www.nets.nihr.ac.uk/projects/hta/082402

This protocol has been prepared in accordance with Standard Protocol Items: Recommendations for Interventional Trials (SPIRIT), a completed checklist is provided as Additional file 1. In accordance with the SPIRIT guidelines, the eTHoS authorship policy is provided in Additional file 2 while the eTHoS participant consent form and patient information leaflet are provided in Additional file 3.

### Ancillary studies

It is recognised, that the value of the study may be enhanced by smaller ancillary studies of specific aspects. Plans for these will be discussed in advance with the PMG. REC approval will be sought for any new proposals, if appropriate.

### Indemnity

The Patient Information Sheet provides the following statement regarding indemnity for negligent and non-negligent harm:

‘We do not expect any harm to come to you by taking part in this study. However, if you are harmed by taking part in this research project, there are no special compensation arrangements. If you are harmed due to someone’s negligence, then you may have grounds for a legal action but you may have to pay for it. Regardless of this, if you wish to complain, or have any concerns about any aspect of the way you have been approached or treated during the course of this study, the normal National Health Service complaints mechanisms (which includes professional indemnity insurance) would be available to you’.

In addition, the universities involved with this study hold and maintain a ‘no fault’ insurance policy. This policy covers all employees of the universities and those working under their direction.

### Data sharing and preservation

The applicants will comply with the data sharing and preservation guidance. The trial statistician (in collaboration with the CI) will manage access rights to the data set. Prospective new users must demonstrate compliance with legal, data protection and ethical guidelines before any data are released. We anticipate that anonymised trial data will be shared with other researchers in the future to enable meta-analyses.

### Publication

The success of the study depends entirely on the wholehearted collaboration of a large number of participants, as well as clinicians, including colorectal surgeons and ROs. For this reason, chief credit for the study will be given, not to the committees or central organisers, but to all those who have collaborated in the study. The results of the study will be reported first to study collaborators. The main report will be drafted by the project management group and circulated to all clinical coordinators for comment. The final version will be agreed by the TSC before submission for publication, on behalf of all the eTHoS collaborators.

To safeguard the integrity of the main trial, reports of ancillary or satellite studies will not be submitted for publication without prior agreement from the PMG.

We intend to maintain interest in the study by publication of eTHoS newsletters at intervals for participants, staff and collaborators. Once the main report has been published, a lay summary of the findings will be sent in a final eTHoS Newsletter to all involved in the trial.

## Trial status

The first participant was recruited in January 2011 and the trial is currently open to recruitment in 29 UK centres.

## Electronic supplementary material

Additional file 1: **SPIRIT checklist.** (PDF 134 KB)

Additional file 2: **eTHoS Authorship Policy.** (PDF 150 KB)

Additional file 3: **Study Participant Consent Form and Patient Information Leaflet.** (PDF 378 KB)

## Abbreviations

AUC: area under the curve
BID: twice a day
BNF: British National Formulary
CEAC: cost-effectiveness acceptability curve
CHaRT: Centre for Healthcare Randomised Trials
CI: chief investigator
CRF: case report form
DCE: discrete choice experiment
DMC: data monitoring committee
EQ-5D: EuroQol Group’s 5 Dimension Health Status Questionnaire
eTHoS: either Traditional haemorrhoidectomy or Stapled Haemorrhoidopexy
GCP: good clinical practice
GP: general practitioner
HALO: haemorrhoidal artery ligation operation
HES: hospital episode statistics
HTA: Health Technology Assessment
ISD: Information Statistics Division
ISRCTN: International Standard Randomised Controlled Trial Number
IT: information technology
MRC: Medical Research Council, UK
NCT: National Clinical Trial
NHS: National Health Service
NIHR: National Institute Health Research, UK
NRES: National Research Ethics Service
OD: once a day
PEDW: Patient Episode Database Wales
PI: principal investigator
PMG: project management group
PRO: patient reported outcome
PSSRU: Personal Social Services Research Unit
QALY: Quality Adjusted Life Year
QID: four times a day
RBL: rubber band ligation
RCT: randomised controlled trial
REC: research ethics committee
RO: recruitment officer
SAE: serious adverse event
SAP: statistical analysis plan
SD: standard deviation
SF-36: Short Form Health Survey 36 Question Survey
SH: stapled haemorrhoidopexy
SHSC: Scottish Health Service Costs
TH: traditional haemorrhoidectomy
TSC: trial steering committee
UK: United Kingdom
UKCRC: United Kingdom Clinical Research Collaboration
VAS: visual analogue score
WTP: willingness to pay.

## References

[1] **NHS Information Centre. UK: NHS, 2006/07**. [http://www.hesonline.nhs.uk/Ease/servlet/ContentServer?siteID=1937&categoryID=215]

[2] Thomson WHF (1975). The nature of haemorrhoids. Br J Surg.

[3] Lacerda-Filho A, Da Silva RG (2005). Stapled haemorrhoidectomy: present status. Arq Gastroenterol.

[4] Shao WJ, Li GC, Zhang ZH, Yang BL, Sun GD, Chen YQ (2008). Systematic review and meta-analysis of randomized controlled trials comparing stapled haemorrhoidopexy with conventional haemorrhoidectomy. Br J Surg.

[5] Jayaraman S, Colquhoun PH, Malthaner RA (2007). Stapled hemorrhoidopexy is associated with a higher long-term recurrence rate of internal hemorrhoids compared with conventional excisional hemorrhoid surgery. Dis Colon Rectum.

[6] Burch J, Epstein D, Baba-Akbari A, Weatherly H, Fox D, Golder S, Jayne D, Drummond M, Woolacott N (2008). Stapled haemorrhoidectomy (haemorrhoidopexy) for the treatment of haemorrhoids: a systematic review and economic evaluation. Health Technol Assess.

[7] Thaha MA, Campbell KL, Kazmi SA, Irvine LA, Khalil A, Binnie NR, Hendry WS, Walker A, Staines HJ, Steele RJ (2009). Prospective randomised multi-centre trial comparing the clinical efficacy, safety and patient acceptability of circular stapled anopexy with closed diathermy haemorrhoidectomy. Gut.

[8] Shanmugam V, Muthukumarasamy G, Cook JA, Vale L, Watson AJM, Loudon MA (2010). Randomised controlled trial comparing rubber band ligation with stapled haemorrhoidopexy for grade II circumferential haemorrhoids: Long term results. Colorectal Dis.

[9] Shanmugam V, Watson AJM, Chapman AD, Binnie NR, Loudon MA (2005). Pathological audit of stapled haemorrhoidopexy. Colorectal Dis.

[10] Jorge JMN, Wexner SD (1993). Etiology and management of fecal incontinence. Dis Colon Rectum.

[11] Taves DR (1973). Minimisation: a new method of assigning patients to treatment and control groups. Clin Pharmacol Ther.

[12] Ryan M, Watson V, Amaya-Amaya M (2003). Methodological issues in the monetary valuation of benefits in healthcare. Expert Rev Pharmacoecon Outcomes Res.

[13] Louviere J, Hensher DA, Swait JD (2000). Stated Choice Methods: Analysis And Application.

[14] Barton GR, Sach TH, Jenkinson CMJ, Avery AJ, Muir KR (2009). Lifestyle interventions for knee pain in overweight and obese adults aged ≥45: economic evaluation of randomised controlled trial. BMJ.

[15] **ISD Scotland**. [http://www.isdscotland.org]

[16] **Department of Health The NHS Mandate: Reference document for policy impact assessment**. [http://webarchive.nationalarchives.gov.uk/20130107105354/http://dh.gov.uk/en/publicationsandstatistics/publications/publicationspolicyandguidance/index.htm]

[17] Philips Z, Ginnelly L, Sculpher M, Claxton K, Golder S, Riemsma R, Woolacoot N, Glanville J (2004). A review of guidelines for good practice in modelling in economic evaluation. Health Technol Assess.

[18] Ross NP, Hildebrand DR, Tiernan JP, Brown SR, Watson AJM (2012). Haemorrhoids: 21st century management. Colorectal Dis.

[19] Tiernan J, Hind D, Watson A, Wailoo AJ, Bradburn M, Shephard N, Biggs K, Brown S (2012). The HubBLe trial: haemorrhoidal artery ligation (HAL) versus rubber band ligation (RBL) for haemorrhoids. BMC Gastroenterol.

[20] Lehur P: **Randomized Controlled Trial Comparing Transanal Doppler-guided Arterial Ligation With Mucopexy and Stapled Haemorrhoidopexy (LIGALONGO).** Clinical Trials.gov. [http://clinicaltrials.gov/show/NCT01240772]

[21] Didnee A-S, Lehur P, For the Ligalongo Group (2013). Multicentre “LigaLongo” RCT comparing DG haemorrhoidal artery ligation/mucopexy and stapled anopexy: a cost-effectiveness analysis [abstract]. Colorectal Dis.

[22] Edwards PJ, Roberts I, Clarke MJ, DiGuiseppi C, Wentz R, Kwan I, Cooper R, Felix LM, Pratap S (2009). Methods to increase response to postal and electronic questionnaires. Cochrane Database Syst Rev.

