# Supplementary material for: A pragmatic, multicentre, randomised controlled trial comparing stapled haemorrhoidopexy to traditional excisional surgery for haemorrhoidal disease (eTHoS): study protocol for a randomised controlled trial
Source: Trials. 2014 Nov 11;15:439. doi: 10.1186/1745-6215-15-439 (PMC4289313; doi:10.1186/1745-6215-15-439)
Supplement: Supplementary file 2 — Additional file 2: eTHoS Authorship Policy. (PDF 150 KB) [file 13063_2014_2363_MOESM2_ESM.pdf]

APPENDICES

Additional file 2

Appendix 1 eTHoS AUTHORSHIP POLICY

1. PRINCIPLES OF AUTHORSHIP

The following principles of authorship have been derived from editorial publications from leading journals (see references) and are in accordance with the rules of the International Committee of Medical Journal Editors.

a. Group authorship

Group authorship will be appropriate for some publications, such as main reports. This will apply when the intellectual work underpinning a publication 'has been carried out by a group, and no one person can be identified as having substantially greater responsibility for its contents than others'[1]. In such cases the authorship will be presented by the collective title - The eTHoS Study Group - and the article should carry a footnote of the names of the people (and their institutions) represented by the corporate title. In some situations one or more authors may take responsibility for drafting the paper but all group members qualify as members; in this case, this should be recognised using the by line 'Jane Doe and the Trial Group',2. Group authorship may also be appropriate for publications where one or more authors take responsibility for a group, in which case the other group members are not authors but may be listed in the acknowledgement (the by line would read 'Jane Doe for the Trial Group')[2].

b. Individual authorship

Other papers, such as describing satellite studies, will have individual authorship. In order to qualify for authorship an individual must fulfil the following criteria1:

i. Each author should have participated sufficiently in the work represented by the article to take public responsibility for the content.

ii. Participation must include three steps:

- conception or design of the work represented by the article OR analysis and interpretation of the data OR both; AND
- drafting the article or revising it for critically important content; AND

- final approval of the version to be published.

Participation solely in the collection of data is insufficient by itself. Those persons who have contributed intellectually to the article but whose contributions do not justify authorship may be acknowledged and their contribution described[1].

c. Determining authorship

Tentative decisions on authorship should be made as soon as possible[1]. These should be justified to, and agreed by, the Project Management Group. Any difficulties or disagreements will be resolved by the Steering Committee.

PUBLICATION ARISING FROM eTHoS TRIAL – OPERATIONALISING AUTHORSHIP RULES

We envisage two types of report (including conference presentations) arising from the eTHoS study and its associated projects:

i. Reports of work arising from the main eTHoS study - If all grant-holders and research staff fulfil authorship rules, group authorship should be used under the collective title of 'The eTHoS Study Group'; if one or more individuals have made a significant contribution above and beyond other group members but where all group members fulfil authorship rules, authorship will be attributed to 'Jane Doe and the eTHoS Study Group'.

ii. Reports of satellite studies and subsidiary projects - Authorship should be guided by the authorship rules outlined in Section 1 above. Grant-holders and research staff not directly associated with the specific project should only be included as authors if they fulfil the authorship rules. Grant-holders and research staff who have made a contribution to the project but do not fulfil authorship rules, should be recognised in the Acknowledgement section. The role of the eTHoS Study Group in the development and support of the project should be recognised in the Acknowledgement section. The lead researcher should be responsible for ratifying authorship with the Project Management Group.

For reports which specifically arise from the eTHoS Study but where all members do not fulfil authorship rules (for example, specialist sub-study publications), authorship should be attributed to 'Jane Doe for the eTHoS Study Group'. If individual members of the group are dissatisfied by a decision, they can appeal to the Management Group for reconciliation. If this cannot be achieved, the matter should be referred to the Steering Group.

b. Quality assurance

Ensuring quality assurance is essential to the good name of the trial group. For reports of individual projects, internal peer review among members of the Project Management Group is a requirement prior to submission of papers. All reports of work arising from the eTHoS Study including conference abstracts should be peer reviewed by the Project Management Group.

The internal peer review for reports of work arising from eTHoS is mandatory and submission may be delayed or vetoed if there are serious concerns about the scientific quality of the report. The Project Management Group will be responsible for decisions about submission following internal peer review. If individual members of the group are dissatisfied by decisions, the matter may be referred to the Steering Group.

The Project Management Group undertakes to respond to submission of articles for peer review at the Project Management Group Meeting following submission (assuming the report is submitted to the trial secretariat in Aberdeen at least two weeks prior to the meeting).

REFERENCES

1. Huth EJ: **Guidelines on authorship of medical papers.** *Ann Intern Med* 1986, **104:**269-274.
2. Glass RM: **New information for authors and readers. Group authorship, acknowledgements and rejected manuscripts.** *JAMA* 1992, **268:**99.
