# Supplementary material for: A pragmatic, multicentre, randomised controlled trial comparing stapled haemorrhoidopexy to traditional excisional surgery for haemorrhoidal disease (eTHoS): study protocol for a randomised controlled trial
Source: Trials. 2014 Nov 11;15:439. doi: 10.1186/1745-6215-15-439 (PMC4289313; doi:10.1186/1745-6215-15-439)
Supplement: Supplementary file 3 — Additional file 3: Study Participant Consent Form and Patient Information Leaflet. (PDF 378 KB) [file 13063_2014_2363_MOESM3_ESM.pdf]

Additional file 3

Appendix 2: Study Participant Consent Form

STUDY CONSENT FORM

eTHoS: either Traditional Haemorrhoidectomy or

Stapled Haemorrhoidopexy for Haemorrhoidal Disease

Participant Study No

Please tick

ALL boxes

↓

By signing this form and ticking each box I agree that I have:

- been given the Information Sheet about the study (Version 2.1, dated Dec 2011) ☐
- had the opportunity to discuss the study ☐
- received satisfactory answers to questions ☐
- been given enough information about the study ☐

I understand that:

- my participation is voluntary and taking part in the study may not benefit my own health ☐
- I am free to withdraw from the study at any time without having to give a reason ☐
- if I withdraw, this will not affect my medical care or legal rights ☐
- I may be contacted in the future for long term follow-up ☐

I agree that relevant sections of my medical notes and data collected during the study may be looked at by individuals from the University of Aberdeen, from regulatory authorities or from the NHS Board or Trust, where it is relevant to my taking part in this research. Information relevant to the eTHoS study may be collected from my hospital and NHS records, including Office of National Statistics (ONS) and NHS central registers. ☐

I agree that relevant data and my contact details will be held confidentially and securely by the study office in Aberdeen, and may be subject to audit and monitoring by regulatory authorities, without breaching data confidentiality ☐

I agree that my family doctor (GP) and my hospital consultant may be told that I am taking part in this study ☐

I agree to take part in the eTHoS study ☐

Your signature (participant) ______________________________

Your name in block capitals ______________________________

Date ______________________________

For office use only

I confirm that I have explained to the person named above, the nature and purpose of the eTHoS study and the procedures involved

Signature Date

eTHoS Study Office, Centre for Healthcare Randomised Trials (CHaRT),
Health Services Research Unit, University of Aberdeen, Foresterhill, Aberdeen AB25 2ZD l
Tel 01224 438170; Fax 01224 438165; Email: ethos@abdn.ac.uk

Copies: White sent to researchers in Aberdeen; pink to participant, green to site file and yellow to be filed with hospital notes.

either Traditional Haemorrhoidectomy
or Stapled Haemorrhoidopexy for
Haemorrhoidal Disease

The eTHoS Study

(either Traditional Haemorrhoidectomy or Stapled Haemorrhoidopexy)

PATIENT INFORMATION LEAFLET

INVITATION TO TAKE PART

You are being invited to take part in a research study. We would like you to take a little time to decide if you would like to take part. Before you do decide, it is important that you understand why the research is being done and what taking part will involve. Please take time to read this information leaflet carefully and you can discuss it with friends, relatives and your GP. If the information is not clear or if you would like more information, please ask us. Contact details are at the end of the leaflet.

WHY HAVE I BEEN INVITED TO TAKE PART?

You have been chosen because you are suitable for this study. You have haemorrhoids and you have been told that surgical treatment is required to improve your symptoms. We hope that approximately 800 patients will take part and complete this study in the UK.

DO I HAVE TO TAKE PART?

No and you do not have to decide today. You have been given this information sheet to take home and you can read it as many times as you wish and ask as many questions as you need to before you make a decision.

If you decide that you do not want to take part in the study now or wish to withdraw at a later stage, this will not affect the standard of care you receive.

If you decide to take part, you will be asked to sign a “consent form” before your participation in the study begins. However, even after you have signed this form, you are still free to withdraw at any time and without giving a reason.

BACKGROUND TO THE CONDITION

Haemorrhoids are swellings around the back passage which can cause bleeding, pain and itching and can protrude. When they become enlarged, surgery is often advised as it is an effective way to control the symptoms.

There are two major surgical treatments for haemorrhoids; “traditional” surgery and a surgical treatment in which the haemorrhoids are “stapled”.

What is the current (‘traditional’) surgical treatment for haemorrhoids?

Traditional surgery involves removal of the swellings in order to improve symptoms. This traditional approach is effective; however it is often associated with immediate post operative pain.

What is the “stapled” surgical treatment?

Newer surgical techniques include “stapled” treatment. The surgery involves cutting away a ring or ‘donut’ of tissue above the swellings and special staples are used to join the tissue again. Its advantages include a possible reduction of pain immediately after surgery, shorter operating time and hospital stay and a quicker return to work. However, over the longer term patients who have this type of surgery may be more likely to experience haemorrhoids again and need further surgery.

Both surgical treatments are commonly performed as a hospital day-case or inpatient admission by colorectal surgeons and, in general, both are successful in improving patients’ symptoms.

WHAT IS THE PURPOSE OF THE STUDY?

At present there is no evidence that tells us which of these types of surgery is best for patients like you who have haemorrhoids that require surgery. The eTHoS study will investigate which of the two surgeries is the best surgical treatment for different kinds of patients.

HOW WILL WE DO THIS?

Patients who agree to take part in the eTHoS study will be divided into two separate treatment groups (see below). The type of treatment patients receive will depend on

which group they are in. The researchers will then compare the general health and quality of life of the people in these two treatment groups over the same amount of time.

Group number	Group name	Group treatment
Group 1	Traditional surgical treatment group	This group will receive traditional surgery for haemorrhoidal disease
Group 2	Stapled surgical treatment group	This group will receive stapled surgery for haemorrhoidal disease

The treatment group that you are put in will be decided at random. This means that neither you nor the doctors will be able to decide which surgical treatment you will receive. This method is used to make sure that the groups of patients who receive the two types of treatment are very similar. Because we do not know which treatment is best, everyone has an equal (50/50) chance of receiving the treatment that is shown to be most effective once the study is completed. To take part in this study you must be happy to be in either of the treatment groups.

HAVE ANY STUDIES LIKE THIS BEEN DONE BEFORE?

Yes. There are several studies that have shown that haemorrhoidal symptoms improve after either type of surgery. However, many of the studies did not follow patients up for long enough afterwards and there is still uncertainty about which type of surgery is most likely to be followed by complications or by more haemorrhoids and a need for further surgery.

Therefore, the eTHoS study is important because it will be the first major study to address all these issues. By participating in the eTHoS study you will be helping to improve our knowledge and this will help us to treat patients with haemorrhoids in the future.

WHAT WILL HAPPEN NEXT?

1) *You are being asked to consider taking part in the study*

You will have been given this information sheet by your surgeon. Your surgeon will be able to answer any questions and discuss with you any aspect of the study during your consultation appointment. During your appointment you will be asked if you would like to take part in the study or if you need more time to consider your decision. You can keep this information sheet and can re-read through the information at home in detail.

2) *What will happen if I am ready to make a decision?*

If you are happy to agree to take part in the study during your appointment, you will be asked to sign a consent form. You will then be asked to complete a questionnaire about your health in general and your haemorrhoidal symptoms such as pain and incontinence. Each questionnaire should take approximately 15 - 20 minutes to complete. After completion of the questionnaire and prior to your surgery you will be assigned by the telephone or computer system (at random) to one of the two treatment groups. If you wish, your surgeon can inform you of the group you have been placed in, once this is known.

3) What will happen if I am not ready to make a decision today?

You will be approached at your next appointment or when admitted before your operation in order to receive your final response and answer any other questions you may have. If you have any questions before your next appointment please feel free to contact your local research nurse or the central office (contact details are at the end of this leaflet).

4) What happens next?

If you decide to take part, you will be placed on the surgery waiting list for the type of surgery you have been randomised to (either traditional or stapled surgery). You are likely to undergo your surgery within approximately two months (from the time of randomisation) but this will vary in different hospitals. There will be the same time to wait for your operation, whether you join the study or not.

Precise procedures for surgery vary. The surgery may be done as a day case or you may have to stay overnight. You may receive a general or local anaesthetic. Your surgeon will discuss these things with you.

After your surgery you will receive an outpatient clinic appointment to return to the hospital to check how you are getting on. If your haemorrhoidal symptoms are still not adequately controlled you may receive further treatment. You will receive an appointment to come back to hospital whether or not you take part in the trial.

IF I PARTICIPATE IN THE STUDY WHAT WILL HAPPEN AFTER MY SURGERY?

The study will collect information from all the patients who take part in the study for five years after surgery. In research this is called “follow up”. Everyone in the study will be followed up in exactly the same way. If you take part in the study you will return to the clinic at the hospital approximately 6 weeks after your operation. You will also be sent questionnaires from the central co-ordinating office in Aberdeen at approximately 1, 3 and 6 weeks and 12, 24 and 60 months after your operation. You will be sent a reply-paid envelope to return the questionnaire.

ARE THERE POSSIBLE BENEFITS TO ME OF TAKING PART?

You will receive proper health care by your consultant whether you choose to participate in the study or not. There is no guarantee that either type of surgery will be better for you than the other.

eTHoS is an important study because if either surgery is found likely to be more beneficial than the other, this will become the treatment of choice for people with haemorrhoids. By taking part in this study you will be directly helping us to inform the treatment of future patients diagnosed with haemorrhoids that need surgical treatment.

WHAT ARE THE POSSIBLE DISADVANTAGES AND RISKS OF TAKING PART?

1) The Stapled surgical treatment group

The chance of having haemorrhoidal symptoms again in the future may be higher than with traditional surgery.

2) The Traditional surgical treatment group

Potential disadvantages/risks may include more post-operative pain, but this can usually be resolved with pain-reducing drugs [illegible]

Page 4 of 7

At present surgeons do not know if the risk of having a complication after surgery, such as bleeding, requirement of blood transfusion, anal stenosis or fissure, urinary retention, incontinence, wound or pelvic infection is higher with one of these two procedures.

WHAT IF THERE IS A PROBLEM?

We do not believe that by taking part in this study there is any greater risk of harm than if you have surgery outside of the study. If any harm occurs while you are taking part in this research project, you will have all the rights and protection that you normally have as a patient. There are no special compensation arrangements for study participants. If you are harmed due to someone's negligence, then you may have grounds for a legal action but you may have to pay for it. Regardless of this, if you wish to complain, or have any concerns about any aspect of the way you have been approached or treated during the course of this study, the National Health Service complaints mechanisms (which include professional indemnity insurance) would be available to you.

If you have a concern about any aspect of this study, you should ask to speak to the researchers who will answer your questions (eTHoS co-ordinating office 01224 438170). If you remain unhappy and wish to complain formally, you can do this through the NHS Complaints Procedure (or Private Institution). Details can be obtained from the hospital.

In addition to this, you may contact the chairman of the eTHoS Trial Steering Committee through the eTHoS study office. Any complaint you have relating to your participation in the study or any possible harm you might suffer will be addressed.

WHAT HAPPENS WHEN THE RESEARCH STUDY STOPS?

Your colorectal surgeon will continue your care and treatment.

WILL MY TAKING PART IN THE STUDY BE KEPT CONFIDENTIAL?

All information that is collected about you will be kept strictly confidential and will be held securely in accordance with the Data Protection Act. Only certain members of the research team will have access to your information, in order that you can be contacted and sent questionnaires. If you participate in the trial, we will notify your GP, unless you prefer that your GP is not informed. Data for all participants in the study, including those who withdraw, will be kept for a minimum of 10 years. As part of the longer term follow-up, the study office will contact you about five years after your surgery to ask how you are keeping and also check your medical notes to find out, for example, if you have had any further haemorrhoid surgery.

WHAT WILL HAPPEN IF I DON'T WANT TO CARRY ON WITH THE STUDY?

You can withdraw from the study at any time, but you will need to continue attending the colorectal department to have your haemorrhoidal condition monitored, which is part of your standard care. If you become incapacitated during the study, we would withdraw you but all relevant data collected prior to that withdrawal would be retained confidentially.

WHAT IF RELEVANT NEW INFORMATION BECOMES AVAILABLE?

If a new treatment or new information about the two surgical procedures being studied becomes available during the study, you will be made aware of this and you can decide if you would like to continue in the study. You may decide this at any time and your decision will not affect the long-term care. If you decide to continue in the study you will be asked to sign an updated consent form. Also, on receiving new information your research doctor might consider it to be in your best interests to withdraw you from the study. He/she will explain the reasons and arrange for your care to continue. If the study is stopped for any other reason, you will be told why and your continuing care will be arranged.

WHAT WILL HAPPEN TO THE RESULTS OF THE STUDY?

The results of the study will be used to standardise surgical procedures for patients with haemorrhoids. These findings will also be published in scientific journals and presented at scientific meetings. The findings will also be made available to patients through

patient organisations, health information websites that are open to the public and the media where possible and appropriate.

WHO IS ORGANISING AND FUNDING THE RESEARCH?

The study has been designed by UK colorectal surgeons and researchers. Patients will be recruited at different hospitals throughout the UK. The study is being funded by the UK National Institute for Health Research (NIHR), Health Technology Assessment (HTA) Programme.

WHO HAS REVIEWED THE STUDY?

This study has been reviewed and approved by the North of Scotland Research Ethics Committee, the Research and Development Department of your local hospital and the study funder (NIHR HTA programme).

THANK YOU

Patients and doctors rely increasingly on the results of clinical studies, such as eTHoS, to make sure they are making the right decisions about treatment. Thank you for taking the time to read this information leaflet, we hope that it has been helpful in enabling you to decide if you would like participate in the eTHoS study.

FURTHER INFORMATION AND CONTACT DETAILS

Central Office contact details:

eTHoS Study Office

Centre for Healthcare Randomised Trials (CHaRT)

Health Services Research Unit

University of Aberdeen

3rd Floor, Health Sciences Building

Foresterhill<br>*Aberdeen AB25 2ZD*<br>*Telephone: 01224 438170*<br>*Email:ethos@abdn.ac.uk*
**Local contact details:**
